# Cathepsin B in programmed cell death machinery: mechanisms of execution and regulatory pathways

**DOI:** 10.1038/s41419-023-05786-0

**Published:** 2023-04-08

**Authors:** Zhen Xie, Mengyuan Zhao, Chengxiang Yan, Wei Kong, Fei Lan, Narengaowa, Shuxuan Zhao, Qinghu Yang, Zhantao Bai, Hong Qing, Junjun Ni

**Affiliations:** 1 Key Laboratory of Molecular Medicine and Biotherapy, Department of Biology, School of Life Science, Beijing Institute of Technology, 100081 Beijing, China; 2 Research Center for Resource Peptide Drugs, Shaanxi Engineering and Technological Research Center for Conversation and Utilization of Regional Biological Resources, Yan’an University, Yan’an, 716000 China; 3 Yan’an Key Laboratory for Neural Immuno-Tumor and Stem Cell and Engineering and Technological Research Center for Natural Peptide Drugs, Yan’an, 716000 China

**Keywords:** Cell biology, Molecular biology

## Abstract

Cathepsin B (CatB), a cysteine protease, is primarily localized within subcellular endosomal and lysosomal compartments. It is involved in the turnover of intracellular and extracellular proteins. Interest is growing in CatB due to its diverse roles in physiological and pathological processes. In functional defective tissues, programmed cell death (PCD) is one of the regulable fundamental mechanisms mediated by CatB, including apoptosis, pyroptosis, ferroptosis, necroptosis, and autophagic cell death. However, CatB-mediated PCD is responsible for disease progression under pathological conditions. In this review, we provide an overview of the critical roles and regulatory pathways of CatB in different types of PCD, and discuss the possibility of CatB as an attractive target in multiple diseases. We also summarize current gaps in the understanding of the involvement of CatB in PCD to highlight future avenues for research.

## Facts

- CatB is involved in multiple kinds of programmed cell death.
- CatB can work in extralysosomal space.
- Inhibitors of CatB are highly effective in many types of PCD-associated diseases.

## Open questions

- Is the extralysosomal location of CatB precisely regulated by potential transporters?
- What is the substrate of CatB in NLRP3 inflammasome activation?
- Does CatB differentially regulated in specific PCD?
- Can CatB induce PCD in lysosomes without translocation?
- How to monitor the relationship between CatB and lysosome membrane integrity?

## Introduction

Cathepsins are endopeptidase found in most cells, which takes part in cell autolysis and self-digestion of tissues. Based on their structure and catalytic type, cathepsins are classified into serine (cathepsins A and G), aspartic (cathepsins D and E), and of cysteine cathepsins (cathepsins B, C, F, H, K, L, O, S, V, W, and X) which is the largest cathepsin family [1]. Because of their overlapping in substrate specificities, cysteine cathepsin networks are able to compensate for the loss of function of individual enzymes. CatZ regulates the acquired tumor-promoting functions of lesions deficient in both CatB and CatS as the compensatory protease [2]. However, double-knockout mice (CatB−/− CatL−/−) died shortly after birth of brain atrophy [3]. It is suggested that not all cathepsins, but only several, may compensate for each other in vivo.

Cathepsin B (CatB, EC 3.4.22.1) is the first and currently the best-characterized member of the C1 family of papain-like, lysosomal cysteine peptidases. It is ubiquitously expressed in most cell and tissue types. Increasing evidence of the pathophysiological roles and substrates of CatB has been reported following the establishment of CatB knockout mouse in 2000 [4]. CatB is synthesized as inactive pre-proenzymes and modified in their N‐glycosidically linked oligosaccharide chains with mannose‐6 phosphate residues posttranslational glycosylation in Golgi apparatus, then transferred to endo/lysosomes. The pH drops within the endo/lysosomes, and inactive CatB is processed via autocatalysis or other proteases into a mature, active two‐chain form [1]. Programmed cell death (PCD) is crucial for organismal homeostasis. Abnormal regulation of this process is associated with a wide variety of human diseases, including immunological and developmental disorders, neurodegeneration, and cancer [5]. Regulated cell death relies on specialized molecular machinery and differs from classic necrosis which is unregulated cell death caused by overwhelming physical, chemical, or biological factors. PCD is a subset of regulated cell death that includes classical apoptosis in the context of development and tissue homeostasis, and other forms that occur in the microenvironment of exogenous or endogenous perturbations, such as pyroptosis, ferroptosis, necroptosis, and autophagic cell death. There is growing evidence suggesting that CatB is involved in PCD pathways at multiple levels which is responsible for the onset and progression of numerous diseases. Under pathological conditions, not only the expression and activity of CatB are increased, but also lysosomal membrane permeabilization leads to the release of CatB into the cytoplasm, initiating various types of PCD [6]. Cystatins, a superfamily of tight-binding inhibitors of papain-like cysteine peptidases, are widely applied to inhibit CatB activity. Inhibition of CatB has yielded positive results in a number of diseases under both laboratory and clinical conditions [7, 8], mainly by blocking CatB-induced apoptosis and autophagy. Therefore, we summarize the molecular mechanisms and regulatory pathways of CatB in PCD, emphasizing CatB as a dominant execution protease in PCD. In addition, we also review CatB-mediated-PCD involved diseases and discuss the therapeutic strategies via inhibition of CatB activity.

## Cathepsin B

**Fig. 1:**
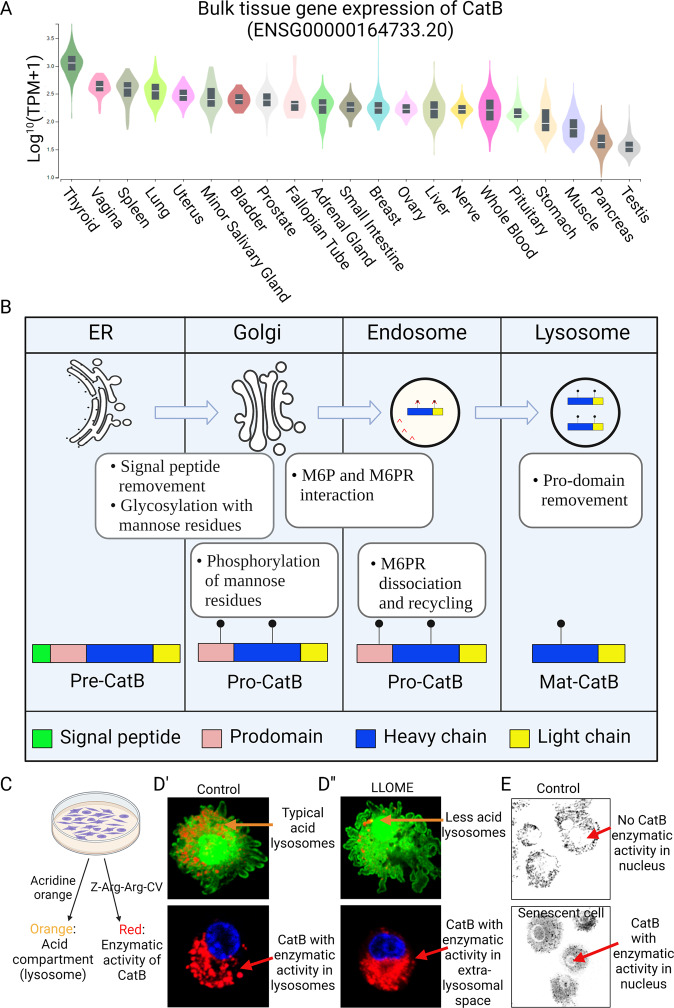
CatB expression, maturation, and cellular distribution. **A** CatB expression in various tissues. The data were obtained from the Genotype-Tissue Expression (GTEx) Project (https://gtexportal.org). Expression values are shown in TPM (transcripts per million), calculated from a gene model with isoforms collapsed to a single gene. **B** CatB maturation and lysosome-sorting pathway. **C** Methods to examine the lysosomal acid condition and CatB enzymatic activity. **D**’ Puncta signals (orange) of lysosomes with the acid environment and puncta signals (red) of CatB with enzymatic activity. **D**” LLOME increases the permeability of the lysosome membrane, resulting in a smaller number of acid lysosomes. LLOME-treated cells showed diffused CatB enzymatic activity. **E** Senescent cells showed CatB enzymatic activity in the nucleus but were not found in control cells.

CatB is found to be ubiquitously expressed in most cell and tissue types (Fig. 1A). It is primarily synthesized as an inactive pre-proenzyme by ribosomes associated with the endoplasmic reticulum, pre-CatB with an N-terminal signal peptide targets this protein to the lumen of endoplasmic reticulum. After removal of the signal peptide (pre) from the endoplasmic reticulum lumen, pro-CatB is delivered to and passed through the different stacks of Golgi apparatus, where pro-CatB is modified in their N-glycosidically linked oligosaccharide chains with mannose-6 phosphate residues. The mannose-6 phosphate and mannose-6 phosphate receptor-mediated transport of pro-CatB from the trans-Golgi network to endo/lysosome. In lysosomes, pro-CatB is further processed via autocatalysis into a mature two-chain form composed of an N-terminal light chain and a C-terminal heavy chain (Fig. 1B) [1]. In addition to the lysosome-sorting pathway, CatB has also been reported to enter the secretory pathway via the default mechanism [9]. In physiological conditions, CatB occurs in the pericellular environment only as their latent precursors. However, enzymatically active extracellular forms of CatB have been found in tumors and plasma [10, 11]. In addition, CatB was detected in the cytosol and nuclear fraction of senescent microglial cells [12, 13], suggesting its extralysosomal function in senescent cell (Fig. 1C–E).

## Cathepsin B in PCD

As a fundamental process of cells, there are complex connections among multiple PCD, and CatB is one of the common regulatory nodal in apoptosis, pyroptosis, ferroptosis, necroptosis and autophagy. CatB showed similar and/or different roles in multiple PCD.

### CatB and apoptosis

**Fig. 2:**
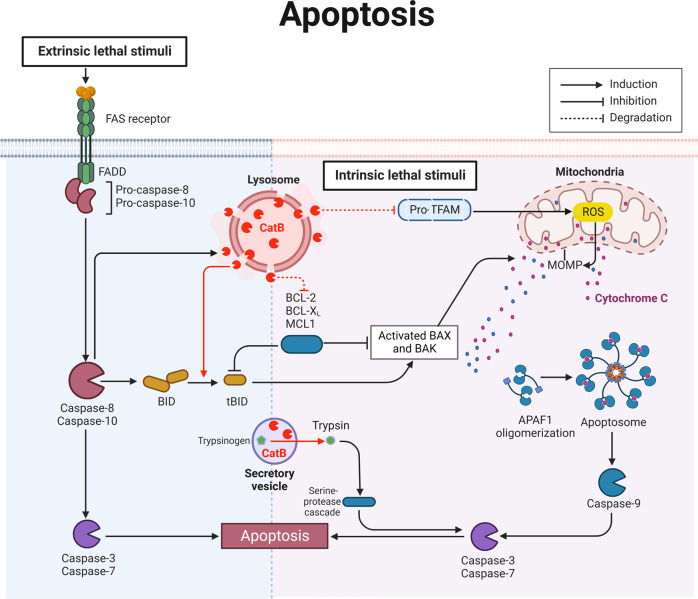
Schematic representation of CatB and its substrates in the core apoptotic machinery. The extrinsic apoptotic pathway is activated by the binding of Fas ligand to the cell surface death receptor (FasR), which induces the activation of the caspase cascade. The intrinsic apoptotic pathway is activated by intracellular stress, which is initiated by BCL-2 family proteins (BAX/BAK). Mitochondria release cytochrome c. The binding of cytochrome c to APAF1 promotes apoptosome assembly, which recruits and activates caspase 9. Notably, leakage of CatB from the lysosome involves in TFAM degradation and Bid truncation, resulting in MOMP. Moreover, CatB in acidic secretory vesicles is involved in trypsin activation, which regulates the serine protease cascade, resulting in apoptosis. APAF1 apoptotic peptidase activating factor 1, BAK Bcl-2 antagonist/killer 1, BAX Bcl-2 associated X, apoptosis regulator, Bcl-2 B-cell lymphoma 2, Bid BH3-interacting domain death agonist, CatB cathepsin B, Fas apoptosis stimulation fragment, FasR Fas cell surface death receptor, MCL1 myeloid cell leukemia sequence 1, MOMP mitochondrial outer membrane permeabilization, ROS reactive oxygen species, tBid truncated Bid, TFAM mitochondrial transcription factor A.

Apoptosis is the most studied and well-understood form of PCD (Fig. 2). In response to lethal stimuli such as apoptosis stimulation fragment ligand, caspases are activated by cleavage at specific aspartic residues, resulting in the removal of an inhibitory N-terminal domain and the production of a large and a small subunit. Caspase-8 and 10 are cleaved primarily in response to extrinsic signals. Activated caspase-8 induces the release of CatB from the lysosome to the cytosol, where it performs BH3-interacting domain agonist (Bid)-cleavage. This triggers mitochondrial cytochrome c release and subsequent apoptosis [14].

Cellular stress-inducing intrinsic damage, such as DNA damage or increased reactive oxygen species (ROS), eventually converges at the mitochondria where the fate of the cell is decided. The balance between pro-apoptotic and anti-apoptotic B-cell lymphoma (Bcl)-2 family proteins determines whether apoptosis occurs or not. CatB-mediated Bcl-2 degradation reduces the inhibition of apoptotic signaling. Bcl-2-like protein 4 (Bax) and Bcl-2 antagonist/killer 1(Bak) are the main pro-apoptotic executioners that form pores in the mitochondrial outer membrane, which induces mitochondrial outer membrane permeabilization [15]. The pro-apoptotic BH3-only proteins, such as Bid, induce Bax/Bak pore formation, either by directly activating Bax/Bak or passively by sequestering the anti-apoptotic Bcl-2 proteins. Once active Bax/Bak have induced mitochondrial outer membrane permeabilization, pro-apoptotic factors such as cytochrome c, are released from the mitochondria into the cytosol. In the presence of ATP, cytochrome c, apoptotic peptidase activating factor -1, and pro-caspase-9 interact and oligomerize, forming a complex that is known as the apoptosome. The apoptosome allows pro-caspase-9 autoactivation. Active caspase-9 then cleaves caspase-3/7, the executioner, thereby promoting apoptosis [16]. On the other hand, the X-linked inhibitor of apoptosis protein (XIAP) has been studied intensely based on its anti-apoptotic function. The anti-apoptotic function of XIAP is regulated by the second mitochondria-derived activator of caspases, which is released from the mitochondrial intermembrane space upon mitochondrial outer membrane permeabilization [17]. Although XIAP is typically thought of as an antagonist of apoptosis, it has been identified as an essential factor in immune inflammatory signaling to intracellular bacterial infection. For instance, upon cytosolic exposure to Shigella bacteria, XIAP is activated to promote an inflammatory response to clear the infection; However, Shigella has evolved a strategy to escape the innate immune defense by Bid-activation, which in turn potentiates the release of mitochondrial second mitochondria-derived activator of caspases to neutralize XIAP-mediated inflammatory signaling [18].

In experimental pancreatitis, CatB undergoes activation in a secretory, vesicular and acidic compartment where it activates trypsinogen, which in turn activates caspase-3-medicated apoptosis [19]. We found aged microglial CatB leaked to the cytosol and degraded Pro-mitochondrial transcription factor A [12], which promotes ROS production. ROS may inhibit the enzymatic activity of cystatin C [20], an endogenous inhibitor of CatB, which then liberates CatB in a positive feedback pattern.

### CatB and pyroptosis

**Fig. 3:**
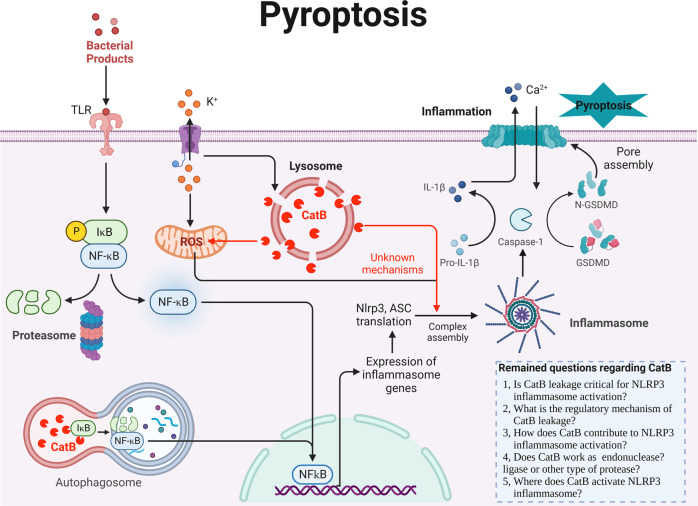
Schematic representation of CatB and its substrates in the core pyroptosis machinery. Activation of NLRP3 inflammasome in macrophages requires two steps: priming and activation. The priming step is provided by inflammatory stimuli such as TLR agonists, which induce NF-κB-mediated NLRP3 and pro-IL-1β expression. The activation step is triggered by PAMPs and DAMPs. Upon stimulation, NLRP3 oligomerizes and recruits ASC through homotypic PYD-PYD interactions and leads to helical ASC filament formation. Assembled ASC recruits caspase 1 and enables proximity-induced caspase 1 self-cleavage and activation, which in turn cleaves pro-IL-1β and GSDMD. GSDMD inserts into the membrane, forming pores and inducing pyroptosis. It has been demonstrated that CatB is a critical factor for NLRP3 inflammasome activation but the precise molecular mechanisms and cellular space are unclear. The remained questions regarding CatB are listed at the right-bottom of the figure. CatB cathepsin B, DAMPs damage-associated molecular patterns, GSDMD gasdermin, IL interleukin, IκB I kappa B kinase, NF-κB nuclear factor kappa-light-chain-enhancer of activated B cells, NLRP3 NACHT-, leucine-rich-repeat- (LRR), and pyrin domain (PYD)-containing protein 3, PAMPS pathogen-associated molecular patterns, ROS reactive oxygen species, TLR toll-like receptor.

Pyroptosis is composed of “pyro” and “ptosis”. “Pyro” means fire, indicating the properties of inflammation of pyroptosis, while “ptosis” means falling, which is consistent with other forms of PCD. Canonical pyroptotic death is mediated by inflammasome assembly, which is accompanied by gasdermin cleavage and interleukin (IL)-1β and IL-18 release (Fig. 3). The most extensively studied inflammasome is the NACHT-, leucine-rich-repeat-, and pyrin domain (PYD)-containing protein 3 (NLRP3). The suggested mechanisms of NLRP3 activation include potassium efflux, mitochondrial ROS generation, translocation of NLRP3 to the mitochondria, the release of mitochondrial DNA, and lysosomal destabilization with cathepsins leakage. However, not all of these events are induced by all NLRP3 agonists and the precise mechanism of NLRP3 activation is still debated. In most cell types, NLRP3 must be primed by binding lipopolysaccharide to Toll-like receptor 4, and priming is known to increase cellular expression of NLRP3 through nuclear factor-κB (NFκB) signaling. Once primed, activated NLRP3 nucleates apoptosis-associated speck-like protein containing a CARD (ASC) into prion-like filaments through PYD-PYD interactions. Pro-caspase-1 filaments subsequently form off of the ASC filaments through CARD-CARD interactions, allowing autoproteolytic activation of pro-caspase-1 into caspase-1. This leads to the secretion of IL-1β and IL-18, and cleavage of gasdermin, whose N-terminal domain can oligomerize to form pores in the cell membrane, inducing cell membrane rupture (Fig. 3).

Interestingly, a diverse set of NLRP3 inflammasome activators have been used to show the requirement of CatB for IL-1β production [21]. There is evidence to suggest that lysosomal CatB leakage into the cytosol is critical for NLRP3 inflammasome activation, although the precise molecular mechanisms are still unknown. However, there are some clues to explain the delicate relationship between CatB and NLRP3 inflammasome. For example, (1) CatB involves in the activation of NFκB, which is responsible for the transcriptional regulation of NLRP3 and IL-1β; (2) Mitochondrial ROS is one of the key factors for NLRP3 activation, and we have reported that leaked CatB in the cytosol could induce mitochondrial ROS production [12]; (3) NLRP3 inflammasome was reported to be activated on the dispersed trans-Golgi network where CatB has the chance to interact with NLRP3 inflammasome [22, 23]. (4) A new finding suggested that the vesicles to which NLRP3 is recruited are of endosomal origin [24]. It is reasonable to assume that CatB may activate NLRP3 inflammasome in the endosomes.

NLRP3 has also been linked to necrosis through pyronecrosis. Similar to classical necrosis, pyronecrosis is accompanied by release of the pro-inflammatory cytokine mediator high mobility group box 1 [25]. Although there are significant differences between pyronecrosis and pyroptosis, such as their differential dependence on caspase-1, they are evidently both pathways which respond to pathogens by promoting the inherently pro-inflammatory release of cellular contents [26]. It was reported that human monocytes carrying disease-associated NLRP3 mutations exhibit excessive necrosis-like cell death by a process dependent on ASC and CatB but not on caspase-1 or IL-1β [25]. These evidences suggested that lysosome activity was necessary in pyronecrosis; However, the molecular regulation of CatB on pyronecrosis remains to be studied.

### CatB and ferroptosis

**Fig. 4:**
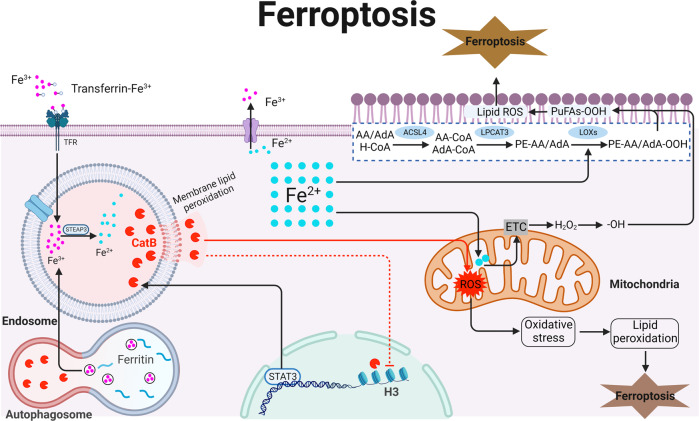
Schematic representation of CatB and its substrates in the core ferroptosis machinery. Ferroptosis is mainly caused by iron-dependent lipid peroxidation. Free Fe^3+^ forms a complex with extracellular transferrin, which binds to a TfR1 on the cell membrane and forms endosomes that are transported in the cell under endocytosis. In the cell, Fe^3+^ is catalyzed into Fe^2+^ by the enzyme STEAP3. Fe^2+^ can be pumped out through ferroportin which is located on the cell membrane. Excess Fe^2+^ generates ROS through Fenton chemical reaction, leading to the continuous accumulation of lipid ROS within the cell and the eventual development of ferroptosis. ROS can also interact with PUFAs on the lipid membrane to form lipid ROS. When a large amount of lipid ROS accumulates in the cell, it causes ferroptosis. Membrane lipid peroxidation of lysosome increases the permeability of the lysosomal CatB, which is capable to promote mitochondrial ROS production and degrades H3 in the nucleus. ACSL4 acyl-coenzyme A synthetase long chain family member 4, CatB cathepsin B, DMT1 divalent metal ion transporter 1, ETC electron transport chain, H3 histone 3, LOX lysyl oxidase, LPCAT3 lysophosphatidylcholine acyltransferase 3, PUFAs polyunsaturated fatty acids, ROS reactive oxygen species, STEAP3 six-transmembrane epithelial antigen of the prostate, TfR1 transferrin receptor 1.

Ferroptosis is characterized by the generation of lipid peroxides by highly expressed unsaturated fatty acids in the cell membrane and the accumulation of lipid peroxides to lethal levels catalyzed by divalent iron or ester oxygenase, thereby inducing cell death (Fig. 4). Iron is an essential micro-element with two oxidation states, Fe^2+^ and Fe^3+^. Fe^3+^ is imported into cells through the membrane protein transferrin receptor 1 and then locates in the endosome. In the endosome, Fe^3+^ is reduced to Fe^2+^ by the ferrireductase activity of six-transmembrane epithelial antigen of prostate 3. Divalent metal transporter 1 mediates the release of Fe^2+^ from the endosome into a labile iron pool in the cytoplasm [27]. Intracellular Fe^2+^ can be oxidized to Fe^3+^ by hephaestin and bound to ferroportin 1, which in turn are transferred out of the cell. Fe^2+^ alters mitochondrial function through the impairment of the electron transport chain complexes. Damaged electron transport chain complexes result in ROS production in mitochondria, leading to oxidative damage to proteins, DNA, and lipids [28]. In addition, ROS can react with the polyunsaturated fatty acids of lipid membranes and induce lipid peroxidation, which accumulates and destroys cell membranes exacerbating the development of ferroptosis (Fig. 4).

Signal transducer and activator of transcription 3 (STAT3) is an oxidative responsive transcriptional factor and is reported to be linked mediation of stress-related ferroptosis. It was demonstrated that genetic blockade of STAT3 limited erastin-induced CatB expression [29]. In contrast, a research results showed overexpression or knockdown of STAT3 had no effects on the expression of CatB [30]. Therefore, STAT3-CatB regulatory axis is stress or disease-dependent in ferroptosis. Maintaining lysosomal integrity and function is crucial for cellular homeostasis. Different forms of stress-induced lysosomal membrane permeabilization resulted in lysosomal-dependent cell death. Using pharmacological inhibitors and genetic models of ferroptosis, lysosomal membrane permeabilization and cytoplasmic leakage of CatB were demonstrated to unleash structural and functional changes in mitochondria and promote cleavage of histone H3 in the nucleus [31]. On the other hand, CatB is demonstrated to involve in the predominantly degradation of albumin in lysosome. Cysteine is a proteogenic amino acid but also needed for the synthesis of glutathione (GSH), which is needed to prevent ferroptosis [32], because GSH is a co-substrate for glutathione peroxidase 4 that could reduce potentially toxic membrane lipid hydroperoxides to non-toxic lipid alcohols. CatB-dependent albumin breakdown followed by export of cystine from the lysosome via the transporter cystinosin fuels the synthesis of GSH [33]. However, catabolism of albumin in lysosome can supply the cell with cysteine need to synthesize GSH while possibly other protective sulfur containing metabolites, block lipid peroxidation, and prevent the onset of ferroptosis.

### CatB and necroptosis

**Fig. 5:**
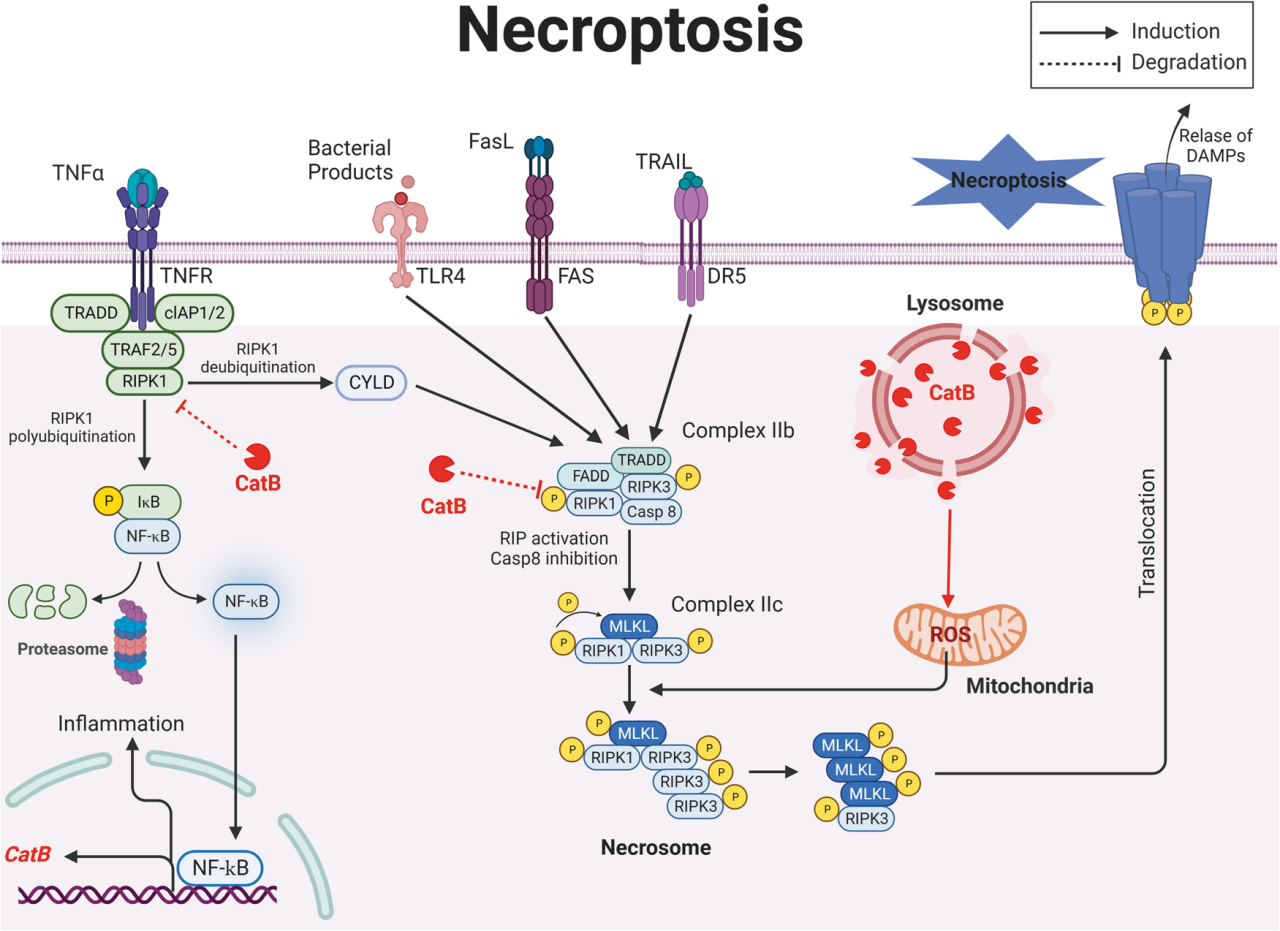
Schematic representation of CatB and its substrates in the core necroptosis machinery. Upon TNF-α stimulation, the activated TNFR interacts TRADD, TRAFs, and RIPK1 and recruits cIAP1/2 to form a plasma membrane-associated complex, resulting in RIPK1 polyubiquitination and NF-κB activation. Deubiquitinated RIPK1 binds to FADD and caspase-8 to form complex IIa, which activates caspase-8 and leads to apoptosis. If caspase-8 activity is blocked, RIPK1 will bind to RIPK3 to form complex IIb, flowed by RIPK3 activation, MLKL phosphorylation, and MLKL membrane translocation resulting in necroptosis through disrupting membrane integrity. Casp caspase, CatB cathepsin B, cIAP1/2 cellular inhibitor of apoptosis protein 1/2, CYLD CYLD lysine 63 deubiquitinase, DAMPs damage-associated molecular patterns, DR5 death receptor 5, FADD Fas associated via death domain, Fas apoptosis stimulation fragment, FasL Fas, apoptosis stimulation fragment ligand, IκB I kappa B kinase, MLKL mixed lineage kinase domain-like, NF-κB nuclear factor kappa-light-chain-enhancer of activated B cells, RIPK receptor interaction protein kinase, ROS reactive oxygen species, TLR toll-like receptor, TNF tumor necrosis factor, TNFR TNF receptor, TRADD TNFR1-associated death domain protein, TRAF TNF receptor associated factor, TRAIL TNF-related apoptosis-inducing ligand.

Necroptosis is a form of regulated cell death, which is induced by ligand binding to tumor necrosis factor family death domain receptors, pattern-recognizing receptors, and virus sensors. The common feature of these systems is the involvement of proteins, containing a receptor interaction protein kinase homology interaction motif mediating recruitment and activation of receptor interaction protein kinase 3, which ultimately activates the necroptosis executioner mixed lineage kinase domain-like. Subsequently, oligomerization of mixed lineage kinase domain-like disrupts the integrity of plasma membranes and causes cell death (Fig. 5). Several lines of evidence demonstrated that lysosomal membrane permeabilization resulting in CatB leakage-mediated mitochondrial ROS plays an indispensable role in the regulation of necroptosis [34, 35]. Besides that, there are few reports on CatB and necroptosis except for direct cleavage of receptor interaction protein kinase 1, demonstrating that CatB pulls the emergency brake on necroptosis [36].

### CatB and autophagy

**Fig. 6:**
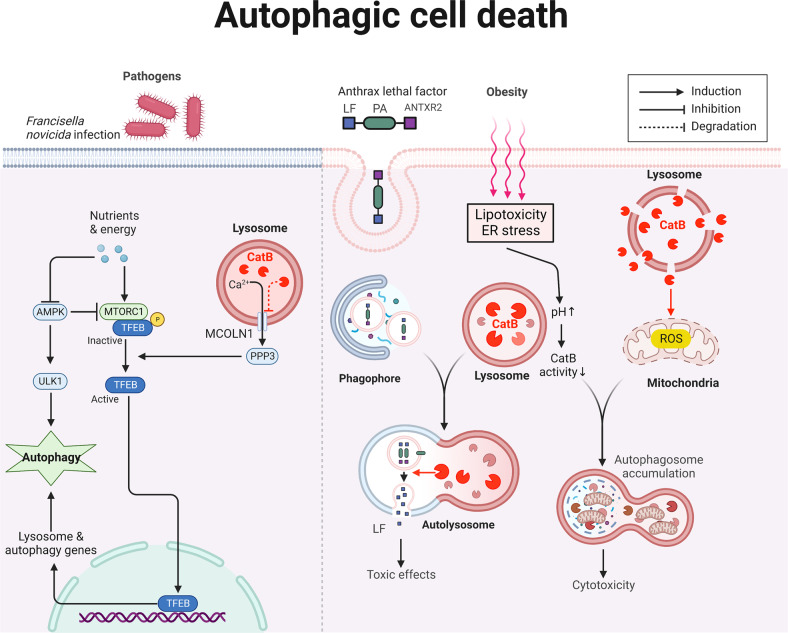
Schematic representation of CatB and its substrates in autophagy. Loss of growth factor stimulation or nutritional inputs like glucose leads to the activation of the ULK complex and subsequently drives phagophore assembly. The phagophore elongates and circularizes to form the autophagosome, which then docks with lysosomes. Under homeostatic conditions, CatB directly cleaves the lysosomal calcium channel MCOLN1 and negatively regulates the efflux of calcium and activation of the PPP3. Inhibition of PPP3 prevents its ability to dephosphorylate and activate TFEB. Dephosphorylated TFEB initiates autophagy. Therefore, CatB works as an apical signal controlling lysosomal dynamics and autophagy. ANTXR2-mediated delivery of LF requires autophagy flux, which is triggered by lysosome fusion and CatB. Obesity-induced lipotoxicity and ER stress, or stressed lysosomal mediated autophagosome accumulation involves CatB activity reduction or lysosomal leakage. AMPK AMP-activated protein kinase, ANTXR2 anthrax toxin receptor 2, CatB cathepsin B, ER endoplasmic reticulum, LF lethal factor, MCOLN1 mucolipin TRP cation channel 1, PPP3 Protein Phosphatase 3, TFEB transcription factor EB, ULK Unc-51 like autophagy activating kinase.

Autophagy is a lysosome-dependent catabolic process characterized by the increased formation of double-membrane autophagosomes for the sequestration of cytoplasmic components and subsequent degradation after autophagosome fusion with lysosomes (Fig. 6). It is generally considered a cell survival/protection mechanism. However, in specific contexts, autophagy accompanies and is required for the activation of other cell death models. In these cases, inhibition of autophagy can prevent cell death, even though cell death is not executed through autophagy.

Under homeostatic conditions, CatB cleaves the calcium channel mucolipin TRP cation channel 1 in the lysosome, maintaining suppression of the transcription factor EB and reducing the expression of lysosomal and autophagy-related proteins [37]. In an infection model of BMDMs with *Francisella movicida*, genetic deletion or pharmacological inhibition of CatB down-regulated mechanistic target of rapamycin activity and prevented cleavage of lysosomal calcium channel TRPML1 and these events drove transcription of lysosomal and autophagy genes via TEEB [38]. Accordingly, CatB provides a checkpoint for homeostatic maintenance of lysosome populations and basic recycling functions in host defense against *Francisella movicida*. Nevertheless, another infection model indicated a contrary mechanic function of CatB in autophagy. Anthrax lethal toxin is a virulence factor secreted by *Bacillus anthracis* and has direct cytotoxic effects on most cells once released into the cytoplasm. The cytoplasmic delivery of the proteolytically active component of lethal toxin, lethal factor, is carried out by the transporter component, protective antigens, which interacts with either of two known surface receptors: anthrax toxin receptor 1 and 2. It was demonstrated that CatB mediates the cytoplasmic delivery of lethal factor by anthrax toxin receptor 2 in parallel with enhanced autophagic flux (Fig. 6), because cells treated with the membrane-permeable CatB inhibitor or CatB-deficient cells had no defect in fusion of LC3-containing autophagic vacuoles with lysosome, autophagic flux was significantly delayed [39]; However, it remains to be addressed how CatB affects this process.

In addition to CatB-mediated autophagy in infection model, it was also reported that increased S-nitrosylation of CatB in both AD mouse and flash-frozen postmortem human AD brains. This posttranslational modification of CatB inhibits its enzymatic activity, blocks autophagic flux, and leads to accumulation of protein aggregates that finally contributes to Alzheimer’s disease pathogenesis [40]. It is worth noting that CatB may involve in autophagy-associated cell death, but lacking evidence of its involvement in autophagy-dependent cell death.

## Cathepsin B and disease

**Fig. 7:**
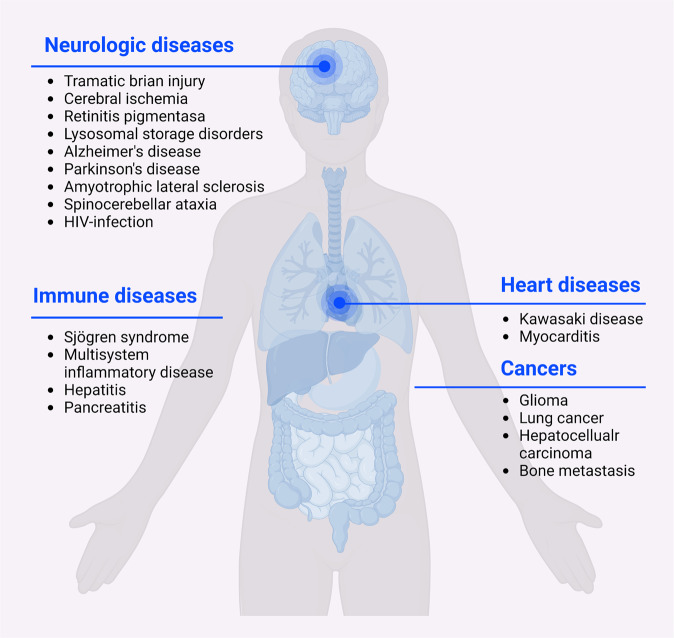
Cathepsin B in diseases. Schematic representation of CatB-mediated cell death in neurologic diseases, immune diseases, cardiac diseases and cancers.

Increased pathological roles and substrates of CatB have been reported in the last decades. Impaired CatB synthesis and activity were shown to be involved in the pathogenesis of multiple diseases (Fig. 7), such as rheumatoid arthritis [41, 42], liver fibrosis [43], traumatic brain injury [44], hypoxia-ischemic brain injury [45], inflammatory pain [46], pancreatitis [47], Alzheimer’s disease [48–50], cancer [51, 52], and COVID-19 infection [53, 54]. Herein, we will review and discuss the role of CatB in PCD during disease progression (Table 1).

**Table 1:** The roles of CatB in PCD during multiple diseases progression.

Diseases	Subjects	CatB expression folds change	CatB activity	Cell death type	CatB pathological function	Reference
Cerebral ischemia	Rat’s model of permanent middle cerebral artery occlusion and in vitro oxygen and glucose deprivation (OGD) model	3	up	Astrocytes and embryonic fibroblast cells	CatB-tBid-mitochondrial apoptotic signaling pathway.	[55]
Cerebral ischemia	Mouse model of permanent middle cerebral artery	7	up	Neurons	Subroutines of necrotic and apoptotic cell death are concomitantly activated by cytoplasmic CatB and the dominant cell death phenotype is determined by the relative speed of each process.	[58, 110]
Cerebral ischemia	2-vessel occlusion with hypotension global brain ischemia rat model	25	–	Pyramidal neurons of the hippocampus and cortex	Extensive CatB release into the cytoplasm results in delayed neuronal death.	[111]
Focal cerebral ischemia	Permanent occlusion of the middle cerebral artery mice model	up	up	Neurons	CatB activates caspase-11 and/or caspase-1 pathways inducing neuronal apoptosis.	[61]
Traumatic brain injury (TBI)	Open-skull controlled cortical impact mouse model	4	up	Neurons	CatB induces programmed cell necrosis and mitochondria-mediated apoptotic pathways.	[56, 62]
Lysosomal storage disorders (LSDs)	Acid sphingomyelinase knockout mouse model; Mucopolysaccharidosis type I mouse model	4–5	up	Cerebellar neurons	Microglial CatB extracellular release promotes neuronal death.	[63, 112]
Parkinson’s disease (PD)	MPTP-induced PD model mice; A53T α-Syn treated BV2 cell	2–4	–	Microglia、dopamine neuron	α-Syn activates NLRP3 inflammasomes through microglial endocytosis and subsequent lysosomal CatB release then inducing cell death	[57]
Alzheimer’s disease (AD)	5xFAD transgenic mouse	–	down	Cerebrocortical primary cell cultures	S-nitrosylating the lysosomal protease CatB inhibits CatB activity and then induces caspase-dependent neuronal apoptosis.	[40]
Alzheimer’s disease	Aβ-treated BV2 cell; peptide chromogranin A treated primary microglia; human amyloid precursor protein (hAPP) model mouse	2–4	up	Microglia; Primary cortical neurons	Aβ/chromogranin-treated microglia causes the release of CatB into the cytoplasm, causing microglia apoptosis, and CatB is released outside the cell, causing neuronal death.	[59, 100, 113]
Retinitis pigmentosa	rd10 mouse carried a missense mutation in the Pde6b gene	–	Decreased in lysosomes and increased in the cytoplasm	Photoreceptor cells	CatB translocates to the cytosol and then promotes apoptosis.	[64]
Hypoxia-ischemia (HI)	Permanent middle cerebral artery occlusion (pMCAO) treated rats	Slightly elevated from 1.5 to 6 h after pMCAO and significantly decreased at 1 day	Significantly increased at 6 h after pMCAO and then gradually decreased	Neonatal cortical neurons	Decreased expression and activity of CatB impairs the autophagic-lysosomal pathway, leading to neuronal death.	[66]
Spinocerebellar ataxia type 6	*Sca6* KI mouse model	KO	–	Purkinje cells (PC)	Lack of CatB expression accelerates mutant Cav2.1 inclusion formation and increases PC loss.	[65]
HIV-1 infection	Neurons cultured with HIV-infected macrophage-conditioned media	2	up	Neuron	CatB–serum amyloid p component (SAPC) interaction triggers neuronal apoptosis.	[69]
Myocardial infarction	Aortic banding mice	3	up	Cardiomyocyte	CatB regulates cardiomyocyte apoptosis via increasing pro-apoptotic proteins Bax and Bid and abating anti-apoptotic protein Bcl-2 expression.	[70, 73, 114, 115]
Viral myocarditis	Intraperitoneal injection of coxsackievirus B3 into mice to induce viral myocarditis	2	up	Cardiomyocyte	CatB activates the NLRP3 inflammasomes promoting cardiomyocyte pyroptosis.	[71]
Type 1 diabetes	Rat insulinoma cell line INS-1E	3	up	Pancreatic β-cell	Lysosomal membrane permeabilization and CatB leakage contribute to the intrinsic pathway of apoptosis.	[116]
Diabetic cardiomyopathy	Streptozotocin injection induced diabetes mouse model; high glucose treated Neonatal rat cardiomyocytes to establish an in vitro model.	1.5	up	Cardiomyocyte	CatB promotes NLRP3-mediated cardiomyocyte pyroptosis.	[72]
Kawasaki disease	Candida albicans cell wall extracts induced mouse model	up	up	Endothelial cell	CatB activates the NLRP3 inflammasomes mediating endothelial cell pyroptosis.	[102]
Sjögren syndrome (SS)	Human salivary gland biopsies	3–4	–	Salivary glands	The release of CatB into the cytoplasm results in apoptosis via activation of caspase 1 and BID-caspase 3 pathways.	[74]
Neonatal onset multisystem inflammatory disease (NOMID)	NOMID patient’s whole blood cell	–	–	Monocyte	CatB contributes to the pyronecrosis of *CIAS1* mutant monocytes.	[75, 76, 117]
Liver injury	Liver fibrosis mice induced by bile duct ligation; free fatty acids treated primary mouse hepatocytes; segmental hepatic ischemia mouse model; TNF-α and actinomycin D treated primary mouse hepatocytes	–	up	Hepatocytes	CatB-tBid-mitochondria-caspase apoptotic signaling pathway.	[43, 78, 79, 118]
Alcohol-associated liver disease (ALD)	Non-alcoholic fatty liver disease patients’ T cell	12	up	Duodenal CD8+ T resident memory (TRM) cells	CatB releases into the cytosol driving the TRM apoptosis.	[119]
Acute pancreatitis	Acinar cells prepared from the pancreas of rats or mice	up	up	Acinar cell	Small amount of CatB in the cytosol activates apoptosis and large amount of CatB shifts the cell death pathway toward necrosis.	[4, 80]
Acute pancreatitis	Intraperitoneal injection of cerulein in mice to induce acute pancreatitis	3	up	Acinar cell	CatB activates the NLRP3 inflammasome and promotes the caspase-1-induced pyroptosis.	[101]

### Neurologic diseases

The cytoplasmic CatB expression has been found to be upregulated in postmortem brain tissue of patients with various neurological diseases, including cerebral ischemia, traumatic brain injury (TBI), and neurodegenerative diseases [40, 55–57]. CatB is sequestered primarily in the lysosomes and vesicles of the regulated secretory pathway, and is rarely found in the cytosol of healthy cells. Numerous researches have reported that lysosomal permeabilization enables the release of CatB into the cytoplasm in response to pathogenic stimuli through in vitro experiments and animal models of disease, including neurons from rats with cerebral ischemia induced by permanent middle cerebral artery occlusion and in vitro oxygen and glucose deprivation model, microglia and neurons treated with Aβ and from AD mice models, dopamine neurons treated with MPTP, cortex and hippocampus neurons from TBI mouse model, etc. [55–59].

Unlike many other lysosomal proteases that are only active at the acidic pH within lysosomes, mature CatB possesses a finite period of proteolytic activity at the neutral pH [60]. Cytosolic CatB cleavage of BID and release of cytochrome C from the mitochondria could induce caspase-dependent apoptosis, which is a common mechanism by which CatB induces cell death in neurological disorders, such as retinitis pigmentosa, (focal) cerebral ischemia, TBI, lysosomal storage disorders, and AD [59, 61–64]. S-nitrosylating CatB, which increases in both 5xFAD transgenic mouse and flash-frozen postmortem human AD brains, showed lower protease activity and compromised autophagic flux induced caspase-dependent neuronal apoptosis in mouse cerebrocortical cultures [65]. A similar mechanism of neuronal cell death caused by the obstruction of autophagosome clearance by low-activity CatB was also found in hypoxia-ischemia [66]. In addition, the deletion of CatB caused Cav2.1 forming inclusions in the lysosomes and induces Purkinje cell death in Spinocerebellar ataxia type 6 mouse model [65]. These studies suggest that CatB exerts neuroprotective effects.

In Parkinson’s disease, microglia endocytosis α-syn results in cytosolic CatB accumulation followed by activation of NLRP3 inflammations, inducing loss of dopaminergic neurons in the substantia nigra [57]. However, the specific substrates and subcellular localization of CatB-initiated NLRP3 activation remain to be revealed. Moreover, only the expression of CatB, rather than Cat H, L, and D, was found to increase in the degenerative neurons of patients with amyotrophic lateral sclerosis (ALS) [67]. Though the exact role of CatB in motor neuron death has not been characterized, Watanabe et al. found that exogenous addition of Cystatin C could protect primary motor neurons derived from ALS model mice by inhibiting CatB activity [8]. Viral infections such as human immunodeficiency virus 1 [68] can also cause neuronal death and cognitive impairment. Human immunodeficiency virus 1 infection of monocyte-derived macrophages, can cross the blood-brain barrier to the central nervous system secreting neurotoxic factors including CatB then trigger neuronal cell apoptosis [68, 69].

### Cardiovascular diseases

CatB expression and protease activity are elevated in several inflammatory heart diseases [70, 71]. High levels of CatB can promote pyroptosis by activating NLRP3 inflammasome in cardiomyocytes of coxsackievirus B3-induced viral myocarditis mice and streptozotocin-induced diabetic cardiomyopathy mice, and in endothelial cells of a mouse model of Kawasaki disease induced by Candida albicans cell wall extracts [72]. Knockout of CatB alleviated apoptosis and pyroptosis of cardiomyocytes in mice with viral myocarditis and myocardial infarction, respectively [71, 73]. Collectively, these evidences suggest that CatB could be a promising therapeutic target for myocarditis.

### Immune disease

As a widely distributed cathepsin in immune cells, CatB is also associated with immune diseases. In Sjögren syndrome, a chronic Kawasaki disease and progressive autoimmune disease characterized by dry mouth and eyes, impaired autophagy has been shown to lead to the release of CatB into the cytoplasm resulting in apoptosis via activation of caspase 1 and BID-caspase 3 pathways [74]. Cold-induced autoinflammatory syndrome 1 gene encodes cryopyrin (named NALP3 and NLRP3), a protein that localizes to the cytosol and functions as pattern recognition receptor. Cryopyrin is one of the main components of NLRP3 inflammasome. Mutations in the cold-induced autoinflammatory syndrome 1 gene induce rapid pyronecrosis of THP-1 monocytic cells and are associated with neonatal-onset multisystem inflammatory disease. The pyronecrosis of cold-induced autoinflammatory syndrome 1 mutant monocytes is independent of caspase1 and IL-1β, but can be blocked by ASC knockdown and CatB inhibition [25, 75–77]. CatB induces hepatocyte death through the CatB-tBid-mitochondrial apoptotic signaling pathway in multiple hepatitis/liver injury models [43, 78, 79]. CatB can also aggravate acute pancreatitis mice induced by intraperitoneal injection of cerulein via activating the NLRP3 inflammasome and promoting caspase-1-induced acinar cell pyroptosis. However, another study showed that treatment of mouse pancreatic acinar cells with low doses of LLOMe resulted in the release of a small amount of CatB into the cytoplasm [80], causing caspase 3-activated apoptotic cell death. At higher doses that lead to large amount of CatB releasing into the cytoplasm, there was in turn a decrease in capsase-3 activation and an increase in lactate dehydrogenase release and RIP-1/RIP-3 interaction suggesting an increase in cell death by necrosis. Moreover, no necrosis was observed when acinar cells of CatB KO mice were treated with high-dose LLOMe [80]. Although the mechanism by which CatB at different cytoplasmic concentrations causes the transformation of cell death pathway remains to be explored, it suggests the variable role of CatB in causing cell death at different stages of disease progression.

### Cancers

CatB expression is elevated in many types of tumor cell and serves as a prognostic and therapeutic marker for multiple cancers [81–83]. Of note, lysosomal cathepsins are often upregulated in cancer cells to meet the increased metabolic demands, and elevated cathepsin expression is associated with increased invasiveness and metastasis. This is significantly different from pathologically stimulated cells in which elevated cytoplasmic CatB induces PCD. In view of the prominent position of its specific functions in cancer cells, there are currently two approaches to directly or indirectly regulate cancer cell death by targeting CatB: (1) Inhibiting the proteolytic enzyme activity of CatB to reduce cancer cell migration, invasion, and proliferation. CatB is involved in cancer metastasis by altering extracellular matrix remodeling and facilitating angiogenesis and promotes invasion and proliferation via induction of vascular endothelial growth factor and MMP-9 [84, 85]. For example, CatB degradation of tenascin-C surrounding neovessels could facilitate neovascular extension resulting in the progression of gliomas [86]. Knockdown of CatB via RNA interference reduced gliomas invasion, growth, and angiogenesis [84]. CatB inhibition by shRNA or CA074 in tumor cells reduced collagen I degradation in vitro and bone metastasis in tumor-bearing animals [87, 88]. (2) Increasing lysosomal permeabilization enables the release of CatB into the cytoplasm to promote tumor cell apoptosis. This is currently the most widely explored in the CatB-targeting treatment strategies of various cancer types, including lung cancer [89], hepatocellular carcinoma [90], and glioma [91]. However, side effects should be evaluated with caution when using these drugs, as lysosomal permeabilization has the potential to cause paracancerous cell death.

## Cathepsin B inhibitors

CatB is involved in multiple types of PCD and diseases. Deletion of CatB has been shown to significantly reduce the disease progression. Therefore, CatB inhibitors have been used in a broad range of disease models to reduce cell death, which demonstrates the important role of CatB in multiple PCD, such as apoptosis, autophagy and pyroptosis, and represents a potential strategy of alleviating the pathogenesis of various diseases. Currently, inhibitors targeting CatB include endogenous peptide inhibitors and both natural and synthetic inhibitors of low molecular weight (Table 2).

**Table 2:** The endogenous and exogenous inhibitors of CatB.

Category	Inhibitor series	Inhibitor	Source	Type of inhibition	Related cells/diseases	Type of cell death	Reference
Endogenous inhibitors	Type I Cystatins	Stefin A	In vivo	Reversible	Keratinoma cells, epithelial, lymphoid/Glioblastoma, breast cancer, head and neck cancer	Apoptosis	[93, 95]
	Type I Cystatins	Stefin B	In vivo	Reversible	Cerebellar granule neurons/Progressive myoclonic epilepsy of the Unverricht-Lundborg (EPM1)	Apoptosis	[60]
	Type II Cystatins	Cystatin C	In vivo	Reversible	Myocardial cell/Viral myocarditis (VMC)	Pyroptosis	[60, 71]
					Motor neuronal cells/Amyotrophic lateral sclerosis (ALS) Dopaminergic neurons/Parkinson’s disease (PD)	Autophagy	[8]
	Type II Cystatins	Cystatin SN	In vivo	Reversible	Cancer cells/Gastric cancer, colorectal cancer (CRC), pancreatic cancer, lung cancer	Autophagy (CRC)	[93, 98]
	Type III Cystatins	H-kininogen	In vivo	Reversible	Microglial cells/Temporal lobe epilepsy (TLE)	/	[98, 108]
Natural inhibitors	Peptidyl aldehydes	Leupeptin	*Streptomyces* strains	Reversible	Microvascular endothelial cells/Scleroderma	/	[105]
		Tokaramide A	*Theonella mirabilis*	Reversible	/	/	[93, 107]
		YM-51084	*Streptomyces* sp. Q21705	Reversible	/	/	[93, 107]
	Aziridinyl peptides	Miraziridine	*Theonella mirabilis*	Irreversible	/	/	[107]
	Epoxysuccinyl peptides	E-64	*Aspergillus japonicus*	Irreversible	Filarial parasite	Apoptosis	[85, 109]
Synthetic inhibitors	Epoxysuccinyl inhibitors	E-64d	Synthesized	Irreversible	Neuronal/Controlled cortical impact (CCI)	Apoptosis	[60]
					Photoreceptor cell/Retinitis pigmentosa	Autophagy	[64, 85]
					Neuronal/Traumatic brain injury (TBI)	Apoptosis	[56]
					Lung cancer cells/Lung cancer	Apoptosis	[120]
		CA074	Synthesized	Irreversible	Liver cells/Non-alcoholic fatty liver disease (NAFLD)	Apoptosis	[79]
					Cortical cell/Focal cerebral ischemia		[61]
					Microglial cells/Alzheimer’s disease (AD)		[100]
					Macrophages, neuronal/HIV-1		[68]
		CA074Me	Synthesized	Irreversible	Acinar cell/Acute pancreatitis (AP)	Apoptosis, pyroptosis	[80, 101]
					Monocytes/Neonatal-onset multisystem inflammatory disease (NOMID)	Pyronecrosis	[75, 76]
					Cardiomyocytes/Cardiac remodeling (heart failure, cardiac hypertrophy	Apoptosis	[73]
					Endothelial cell/Kawasaki disease (KD)	Pyroptosis	[102]
					Retinal pigment epithelial cells/Diabetic retinopathy (DR)	Autophagy	[121]
					Acute myeloid leukemia (AML) cell line THP-1/AML	Apoptosis	[83]
					Mammary epithelial cells/Tumor	Autophagy	[122]
	Aziridines	Aziridine-2,3- dicarboxylate containing inhibitor	Synthesized	Irreversible	Leishmania	Autophagy	[103]
	Nitriles	Azapeptide nitriles	Synthesized	Reversible	/	/	[93]
	/	K11777	Synthesized	Irreversible	Peripancreatic cells/Pancreatic inflammation	/	[104]
	/	Z-Phe-Ala-CH_2_F (Z-FA-fmk)	Synthesized	Irreversible	Neuronal/Alzheimer’s disease (AD)	Apoptosis	[113]
Monoclonal antibodies	/	ProCatB -CDR3H of Herceptin	Synthesized	Antibody	/	/	[107]
	/	mAb 2A2	Synthesized	Antibody	Tumor cell/Tumor	/	[93]

### CatB endogenous inhibitors

Active CatB is generally located in lysosomes and can be detected in various cellular compartments. Although the activity of CatB is reduced after its release into the cytoplasm, any leakage will cause great damage to the cells. Therefore, the endogenous inhibitors of CatB play important roles in the homeostasis of cells, the imbalance between CatB and its endogenous inhibitors is considered to be a hallmark of disease progression [92]. The endogenous inhibitors of CatB include cystatins, thyropins, propeptides, serpins and the general peptidase inhibitor α2-macroglobulin. The most essential one of them is the cystatin superfamily, including evolutionary related proteins expressed in animals, plants, fungi and viruses [93].

The cystatins are divided into three families: type I, II and III. The type I cystatins, which includes cystatin A and B (also known as stefins), is an intracellular protein mainly found in the cytoplasm and nucleus. Stefins are single-chain polypeptide consisting of about 100 amino acids. The type II and III cystatins are secreted proteins. The type II cystatins, such as cystatin C, D, E/M, S and SN, are single-chain polypeptides consisting of about 120 amino acid residues. The activity of CatB in extracellular fluid is mainly regulated by cystatin C. Type III cystatins are multidomain, high molecular mass proteins (60–120 kDa), such as kininogens [60, 94].

Cystatin superfamily is responsible for overseeing the CatB biosynthesis and translocation to lysosomes, regulating excessive cysteine protease activity in cells and tissues [85]. In addition, Stefin A, as a reversible inhibitor, plays a role in apoptosis, controls the proliferation and differentiation of normal keratinoma cells, and protects epithelial and lymphoid from cysteine peptidase produced by invasive pathogens. Stefin A has also been shown to control follicle growth and maturation [60]. It has been reported that low stefin A expression can promote the development of glioblastoma, breast cancer and head and neck cancer. However, CatB and stefin A can form a positive feedback loop during renal cell carcinoma progression in renal cell carcinoma [95]. Aberrant expression of stefin B is also associated with diseases. Compared with benign meningioma, stefin B mRNA and protein expression levels are lower in atypical, whereas the corresponding expression levels of CatB are high [94]. The loss of function mutations in the stefin B gene can cause progressive myoclonic epilepsy of the Unverricht-Lundborg, and the absence of stefin B leads to the increased activity of cysteine cathepsin, inducing apoptosis [60]. Cystatin C can exhibit neuroprotective property through autophagy induction and inhibition of CatB [8]. Cystatin SN regulates proliferation of cancer cells in gastric cancer, pancreatic cancer and lung cancer, and inhibits cell death by regulating autophagy induction and ROS production in colorectal cancer [7]. H-kininogen inhibits bradykinin - induced brain inflammation which is characterized by gliosis [96].

### Exogenous inhibitors of CatB

The group of exogenous inhibitors of CatB includes inhibitors of natural origin that are isolated from animals, plants and microorganisms; small synthetic molecules; neutralizing monoclonal antibodies; anti-sense and siRNA molecules [93].

### Inhibitors of natural origin

CatB inhibitors of natural origin mainly include of peptidyl aldehydes, aziridinyl peptides and epoxysuccinyl peptides. The epoxysuccinyl peptides are the most studied CatB inhibitors of natural origin, and the most well-known member among them is E-64, which was isolated from *Aspergillus japonicus* in 1978. E-64 can induce oxidative stress and apoptosis in various parasites, such as filarial parasite [97]. Aldehyde inhibitor leupeptine, which was isolated from different culture broths of *streptomyces* strains, was shown to ameliorate the harmful effects on endothelial cells in scleroderma [98, 99].

### Synthetic inhibitors

To improve the specificity of CatB inhibitors, another representative epoxysuccinyl inhibitor, CA074 was synthesized with a better selectivity for CatB than E-64. However, the poor membrane permeability of CA074 limits its biological application. CA-074Me was further synthesized and showed strong cellular uptake.

Numerous studies showed that the derivatives of E-64 and CA074 could reduce inflammation and apoptosis in various diseases. For instance, TBI- induced cell death, motor and cognitive dysfunction were attenuated by the use of E-64d [60]. E-64d treatment reduced neuromotor disorders, the loss of brain tissue and neuronal cell in controlled cortical impact mice [60]. Moreover, CA074 could protect cortical structures from ischemic damage in focal cerebral ischemia [61] and abolish the neurotoxic effects caused by Aβ42-activated microglial BV2 cells in AD [100]. CA074ME has been reported to suppress the expression and activity of pro-inflammatory cytokine and further inhibits acinar cell apoptosis and pyroptosis in Acute Pancreatitis [80, 101]. As a potent inhibitor, CA074ME can also be used in the treatment for neonatal-onset multisystem inflammatory disease [75, 76], kawasaki disease [102] and acute myeloid leukemia [83]. Except for the most common epoxysuccinyl inhibitors, aziridine-2,3-dicarboxylate containing inhibitor can induce cell death in Leishmania [103], K11777 is able to improve pain in pancreatic inflammation [104].

Epoxysuccinyl inhibitors are the most studied class of irreversible CatB inhibitors. However, the irreversibility and possible side effects limit their clinical application, which led to the development of reversible inhibitors [93]. A focused library of derivatives of nitroxoline has been reported, which is a potent, selective and reversible CatB inhibitor can alleviate tumor cell invasion [105]. In addition, El-Fakharany et al. revealed the apoptosis-mediating anticancer activity of bovine lactoperoxidase and lactoferrin nanocombinations with Cu and Fe, these novel nanocombinations have high binding affinity to CatB and show a predicted inhibitory effect on it [106].

### Monoclonal antibodies

Monoclonal antibodies have high affinity and specificity for homologous antigens compared to small molecule and peptide-like protease inhibitors. Dai et al. generated a humanized antibody inhibitor with high potency and specificity for human CatB through genetic fusion of propeptide of proCatB into the heavy chain complementarity determining region 3 of Herceptin, which is a drug used for the treatment of breast cancer [107]. Kos et al. reported a recombinant chimeric analog of the murine mAb 2A2, which retained the binding properties of the parental murine antibody and its ability to inhibit CatB activity, mAb 2A2 significantly reduced extracellular matrix degradation and tumor cell invasion in vitro [93].

Overall, chemical inhibition of CatB has been studied in a variety of diseases, includes trauma of cerebral bleeding, Huntington’s disease, excitatory epilepsy, inflammatory pain and neurodegenerative diseases [108]. However, the ideal inhibitor of CatB with good cell permeability, selectivity, efficacy and safety is yet to be developed. Furthermore, due to the compensatory mechanism between other cysteine cathepsins (such as CatX) and CatB observed in cancer and inflammation, long-term use of CatB inhibitors may not prove to be effective in clinical treatment [60, 109].

## Conclusion and perspectives

Major advances have taken place in our understanding of the role of CatB in PCD. Abnormal PCD caused by increased CatB expression and activity is an important trigger for numerous diseases’ pathological development. Therefore, multiple CatB inhibitors have been developed to treat a variety of diseases and have some inspirit outcomes in both laboratory and clinical fields.

In particular, lysosomal leakage of CatB has been reported to be involved in all the mentioned types of PCD and most of the diseases to which CatB-mediated PCD contributes. However, there are still many gaps of the processes and mechanisms underlying lysosomal CatB translocation regulation. And the specific sites of CatB, which leaked into neutral cytoplasmic environments cleavage or hydrolysis substrates, need to be further explored. A better understanding of how lysosomal CatB and mitochondrial redox signals are communicated is not only essential to expand our knowledge of basic biology in PCD, but it will also have important implications in drug development.

## Data Availability

There are no experimental datasets, given that this is a review article that is prepared based on a literature review.

## References

[1] Ni J, Lan F, Xu Y, Nakanishi H, Li X (2022). Extralysosomal cathepsin B in central nervous system: mechanisms and therapeutic implications. Brain Pathol.

[2] Akkari L, Gocheva V, Quick ML, Kester JC, Spencer AK, Garfall AL (2016). Combined deletion of cathepsin protease family members reveals compensatory mechanisms in cancer. Genes Dev.

[3] Felbor U, Kessler B, Mothes W, Goebel HH, Ploegh HL, Bronson RT (2002). Neuronal loss and brain atrophy in mice lacking cathepsins B and L. Proc Natl Acad Sci USA.

[4] Halangk W, Lerch MM, Brandt-Nedelev B, Roth W, Ruthenbuerger M, Reinheckel T (2000). Role of cathepsin B in intracellular trypsinogen activation and the onset of acute pancreatitis. J Clin Invest.

[5] Fuchs Y, Steller H (2011). Programmed cell death in animal development and disease. Cell.

[6] Zamyatnin AA, Gregory LC, Townsend PA, Soond SM (2022). Beyond basic research: the contribution of cathepsin B to cancer development, diagnosis and therapy. Expert Opin Ther Targets.

[7] Liu Y, Yao J (2019). Research progress of cystatin SN in cancer. Onco Targets Ther.

[8] Watanabe S, Hayakawa T, Wakasugi K, Yamanaka K (2014). Cystatin C protects neuronal cells against mutant copper-zinc superoxide dismutase-mediated toxicity. Cell Death Dis.

[9] Kukor Z, Mayerle J, Kruger B, Toth M, Steed PM, Halangk W (2002). Presence of cathepsin B in the human pancreatic secretory pathway and its role in trypsinogen activation during hereditary pancreatitis. J Biol Chem.

[10] Portnoy DA, Erickson AH, Kochan J, Ravetch JV, Unkeless JC (1986). Cloning and characterization of a mouse cysteine proteinase. J Biol Chem.

[11] Moon HY, Becke A, Berron D, Becker B, Sah N, Benoni G (2016). Running-induced systemic cathepsin B secretion is associated with memory function. Cell Metab.

[12] Ni J, Wu Z, Stoka V, Meng J, Hayashi Y, Peters C (2019). Increased expression and altered subcellular distribution of cathepsin B in microglia induce cognitive impairment through oxidative stress and inflammatory response in mice. Aging Cell.

[13] Meng J, Liu Y, Xie Z, Qing H, Lei P, Ni J (2020). Nucleus distribution of cathepsin B in senescent microglia promotes brain aging through degradation of sirtuins. Neurobiol Aging.

[14] Stoka V, Turk B, Schendel SL, Kim TH, Cirman T, Snipas SJ (2001). Lysosomal protease pathways to apoptosis. Cleavage of bid, not pro-caspases, is the most likely route. J Biol Chem.

[15] Heimer S, Knoll G, Schulze-Osthoff K, Ehrenschwender M (2019). Raptinal bypasses BAX, BAK, and BOK for mitochondrial outer membrane permeabilization and intrinsic apoptosis. Cell Death Dis.

[16] Elena-Real CA, Diaz-Quintana A, Gonzalez-Arzola K, Velazquez-Campoy A, Orzaez M, Lopez-Rivas A (2018). Cytochrome c speeds up caspase cascade activation by blocking 14-3-3epsilon-dependent Apaf-1 inhibition. Cell Death Dis.

[17] Flanagan L, Sebastia J, Tuffy LP, Spring A, Lichawska A, Devocelle M (2010). XIAP impairs Smac release from the mitochondria during apoptosis. Cell Death Dis.

[18] Andree M, Seeger JM, Schull S, Coutelle O, Wagner-Stippich D, Wiegmann K (2014). BID-dependent release of mitochondrial SMAC dampens XIAP-mediated immunity against Shigella. EMBO J.

[19] Sendler M, Maertin S, John D, Persike M, Weiss FU, Kruger B (2016). Cathepsin B activity initiates apoptosis via digestive protease activation in pancreatic acinar cells and experimental pancreatitis. J Biol Chem.

[20] Xu Y, Lindemann P, Vega-Ramos J, Zhang JG, Villadangos JA (2014). Developmental regulation of synthesis and dimerization of the amyloidogenic protease inhibitor cystatin C in the hematopoietic system. J Biol Chem.

[21] Hentze H, Lin XY, Choi MS, Porter AG (2003). Critical role for cathepsin B in mediating caspase-1-dependent interleukin-18 maturation and caspase-1-independent necrosis triggered by the microbial toxin nigericin. Cell Death Differ.

[22] Chen J, Chen ZJ (2018). PtdIns4P on dispersed trans-Golgi network mediates NLRP3 inflammasome activation. Nature.

[23] Sharif H, Wang L, Wang WL, Magupalli VG, Andreeva L, Qiao Q (2019). Structural mechanism for NEK7-licensed activation of NLRP3 inflammasome. Nature.

[24] Zhang Z, Venditti R, Ran L, Liu Z, Vivot K, Schurmann A (2022). Distinct changes in endosomal composition promote NLRP3 inflammasome activation. Nat Immunol.

[25] Willingham SB, Bergstralh DT, O’Connor W, Morrison AC, Taxman DJ, Duncan JA (2007). Microbial pathogen-induced necrotic cell death mediated by the inflammasome components CIAS1/cryopyrin/NLRP3 and ASC. Cell Host Microbe.

[26] Ting JP, Willingham SB, Bergstralh DT (2008). NLRs at the intersection of cell death and immunity. Nat Rev Immunol.

[27] Xie Y, Hou W, Song X, Yu Y, Huang J, Sun X (2016). Ferroptosis: process and function. Cell Death Differ.

[28] Gao M, Yi J, Zhu J, Minikes AM, Monian P, Thompson CB (2019). Role of mitochondria in ferroptosis. Mol Cell.

[29] Gao H, Bai Y, Jia Y, Zhao Y, Kang R, Tang D (2018). Ferroptosis is a lysosomal cell death process. Biochem Biophys Res Commun.

[30] Ouyang S, Li H, Lou L, Huang Q, Zhang Z, Mo J (2022). Inhibition of STAT3-ferroptosis negative regulatory axis suppresses tumor growth and alleviates chemoresistance in gastric cancer. Redox Biol.

[31] Nagakannan P, Islam MI, Conrad M, Eftekharpour E (2021). Cathepsin B is an executioner of ferroptosis. Biochim Biophys Acta Mol Cell Res.

[32] Badgley MA, Kremer DM, Maurer HC, DelGiorno KE, Lee HJ, Purohit V (2020). Cysteine depletion induces pancreatic tumor ferroptosis in mice. Science.

[33] Armenta DA, Laqtom NN, Alchemy G, Dong W, Morrow D, Poltorack CD (2022). Ferroptosis inhibition by lysosome-dependent catabolism of extracellular protein. Cell Chem Biol.

[34] Lou J, Wang X, Zhang H, Yu G, Ding J, Zhu X (2022). Inhibition of PLA2G4E/cPLA2 promotes survival of random skin flaps by alleviating Lysosomal membrane permeabilization-Induced necroptosis. Autophagy.

[35] Mulay SR, Honarpisheh MM, Foresto-Neto O, Shi C, Desai J, Zhao ZB (2019). Mitochondria permeability transition versus necroptosis in oxalate-induced AKI. J Am Soc Nephrol.

[36] McComb S, Shutinoski B, Thurston S, Cessford E, Kumar K, Sad S (2014). Cathepsins limit macrophage necroptosis through cleavage of Rip1 kinase. J Immunol.

[37] Man SM, Kanneganti TD (2016). Regulation of lysosomal dynamics and autophagy by CTSB/cathepsin B. Autophagy.

[38] Qi X, Man SM, Malireddi RK, Karki R, Lupfer C, Gurung P (2016). Cathepsin B modulates lysosomal biogenesis and host defense against Francisella novicida infection. J Exp Med.

[39] Ha SD, Ham B, Mogridge J, Saftig P, Lin S, Kim SO (2010). Cathepsin B-mediated autophagy flux facilitates the anthrax toxin receptor 2-mediated delivery of anthrax lethal factor into the cytoplasm. J Biol Chem.

[40] Kim KR, Cho EJ, Eom JW, Oh SS, Nakamura T, Oh CK (2022). S-Nitrosylation of cathepsin B affects autophagic flux and accumulation of protein aggregates in neurodegenerative disorders. Cell Death Differ.

[41] Hashimoto Y, Kakegawa H, Narita Y, Hachiya Y, Hayakawa T, Kos J (2001). Significance of cathepsin B accumulation in synovial fluid of rheumatoid arthritis. Biochem Biophys Res Commun.

[42] Yoshifuji H, Umehara H, Maruyama H, Itoh M, Tanaka M, Kawabata D (2005). Amelioration of experimental arthritis by a calpain-inhibitory compound: regulation of cytokine production by E-64-d in vivo and in vitro. Int Immunol.

[43] Canbay A, Guicciardi ME, Higuchi H, Feldstein A, Bronk SF, Rydzewski R (2003). Cathepsin B inactivation attenuates hepatic injury and fibrosis during cholestasis. J Clin Invest.

[44] Hook G, Jacobsen JS, Grabstein K, Kindy M, Hook V (2015). Cathepsin B is a new drug target for traumatic brain injury therapeutics: evidence for E64d as a promising lead drug candidate. Front Neurol.

[45] Ni J, Wu Z, Peterts C, Yamamoto K, Qing H, Nakanishi H (2015). The critical role of proteolytic relay through cathepsins B and E in the phenotypic change of microglia/macrophage. J Neurosci.

[46] Sun L, Wu Z, Hayashi Y, Peters C, Tsuda M, Inoue K (2012). Microglial cathepsin B contributes to the initiation of peripheral inflammation-induced chronic pain. J Neurosci.

[47] Van Acker GJ, Saluja AK, Bhagat L, Singh VP, Song AM, Steer ML (2002). Cathepsin B inhibition prevents trypsinogen activation and reduces pancreatitis severity. Am J Physiol Gastrointest Liver Physiol.

[48] Hook VY, Kindy M, Reinheckel T, Peters C, Hook G (2009). Genetic cathepsin B deficiency reduces beta-amyloid in transgenic mice expressing human wild-type amyloid precursor protein. Biochem Biophys Res Commun.

[49] Kindy MS, Yu J, Zhu H, El-Amouri SS, Hook V, Hook GR (2012). Deletion of the cathepsin B gene improves memory deficits in a transgenic Alzheimer’s disease mouse model expressing AbetaPP containing the wild-type beta-secretase site sequence. J Alzheimers Dis.

[50] Embury CM, Dyavarshetty B, Lu Y, Wiederin JL, Ciborowski P, Gendelman HE (2017). Cathepsin B improves ss-amyloidosis and learning and memory in models of Alzheimer’s disease. J Neuroimmune Pharmacol.

[51] Palermo C, Joyce JA (2008). Cysteine cathepsin proteases as pharmacological targets in cancer. Trends Pharm Sci.

[52] Yan S, Sloane BF (2003). Molecular regulation of human cathepsin B: implication in pathologies. Biol Chem.

[53] Pislar A, Mitrovic A, Sabotic J, Pecar Fonovic U, Perisic Nanut M, Jakos T (2020). The role of cysteine peptidases in coronavirus cell entry and replication: the therapeutic potential of cathepsin inhibitors. PLoS Pathog.

[54] Padmanabhan P, Desikan R, Dixit NM (2020). Targeting TMPRSS2 and Cathepsin B/L together may be synergistic against SARS-CoV-2 infection. PLoS Comput Biol.

[55] Zhou XY, Luo Y, Zhu YM, Liu ZH, Kent TA, Rong JG (2017). Inhibition of autophagy blocks cathepsins-tBid-mitochondrial apoptotic signaling pathway via stabilization of lysosomal membrane in ischemic astrocytes. Cell Death Dis.

[56] Hook GR, Yu J, Sipes N, Pierschbacher MD, Hook V, Kindy MS (2014). The cysteine protease cathepsin B is a key drug target and cysteine protease inhibitors are potential therapeutics for traumatic brain injury. J Neurotrauma.

[57] Zhou Y, Lu M, Du RH, Qiao C, Jiang CY, Zhang KZ (2016). MicroRNA-7 targets Nod-like receptor protein 3 inflammasome to modulate neuroinflammation in the pathogenesis of Parkinson’s disease. Mol Neurodegener.

[58] Zhang ZB, Li ZG (2012). Cathepsin B and phospo-JNK in relation to ongoing apoptosis after transient focal cerebral ischemia in the rat. Neurochem Res.

[59] Mueller-Steiner S, Zhou Y, Arai H, Roberson ED, Sun B, Chen J (2006). Antiamyloidogenic and neuroprotective functions of cathepsin B: implications for Alzheimer’s disease. Neuron.

[60] Hook G, Reinheckel T, Ni J, Wu Z, Kindy M, Peters C (2022). Cathepsin B gene knockout improves behavioral deficits and reduces pathology in models of neurologic disorders. Pharm Rev.

[61] Benchoua A, Braudeau J, Reis A, Couriaud C, Onteniente B (2004). Activation of proinflammatory caspases by cathepsin B in focal cerebral ischemia. J Cereb Blood Flow Metab.

[62] Luo CL, Chen XP, Yang R, Sun YX, Li QQ, Bao HJ (2010). Cathepsin B contributes to traumatic brain injury-induced cell death through a mitochondria-mediated apoptotic pathway. J Neurosci Res.

[63] Viana GM, Gonzalez EA, Alvarez MMP, Cavalheiro RP, do Nascimento CC, Baldo G, et al. Cathepsin B-associated activation of amyloidogenic pathway in murine mucopolysaccharidosis type I brain cortex. Int J Mol Sci. 2020;21. 10.3390/ijms21041459. 10.3390/ijms21041459 PMC7073069 32093427

[64] Rodriguez-Muela N, Hernandez-Pinto AM, Serrano-Puebla A, Garcia-Ledo L, Latorre SH, de la Rosa EJ (2015). Lysosomal membrane permeabilization and autophagy blockade contribute to photoreceptor cell death in a mouse model of retinitis pigmentosa. Cell Death Differ.

[65] Unno T, Wakamori M, Koike M, Uchiyama Y, Ishikawa K, Kubota H (2012). Development of Purkinje cell degeneration in a knockin mouse model reveals lysosomal involvement in the pathogenesis of SCA6. Proc Natl Acad Sci USA.

[66] Liu Y, Xue X, Zhang H, Che X, Luo J, Wang P (2019). Neuronal-targeted TFEB rescues dysfunction of the autophagy-lysosomal pathway and alleviates ischemic injury in permanent cerebral ischemia. Autophagy.

[67] Kikuchi H, Yamada T, Furuya H, Doh-ura K, Ohyagi Y, Iwaki T (2003). Involvement of cathepsin B in the motor neuron degeneration of amyotrophic lateral sclerosis. Acta Neuropathol.

[68] Zenon-Melendez CN, Carrasquillo Carrion K, Cantres Rosario Y, Roche Lima A, Melendez LM (2022). Inhibition of cathepsin B and SAPC secreted by HIV-infected macrophages reverses common and unique apoptosis pathways. J Proteome Res.

[69] Cantres-Rosario YM, Hernandez N, Negron K, Perez-Laspiur J, Leszyk J, Shaffer SA (2015). Interacting partners of macrophage-secreted cathepsin B contribute to HIV-induced neuronal apoptosis. AIDS.

[70] Ge J, Zhao G, Chen R, Li S, Wang S, Zhang X (2006). Enhanced myocardial cathepsin B expression in patients with dilated cardiomyopathy. Eur J Heart Fail.

[71] Wang Y, Jia L, Shen J, Wang Y, Fu Z, Su SA (2018). Cathepsin B aggravates coxsackievirus B3-induced myocarditis through activating the inflammasome and promoting pyroptosis. PLoS Pathog.

[72] Liu C, Yao Q, Hu T, Cai Z, Xie Q, Zhao J (2022). Cathepsin B deteriorates diabetic cardiomyopathy induced by streptozotocin via promoting NLRP3-mediated pyroptosis. Mol Ther Nucleic Acids.

[73] Wu QQ, Xu M, Yuan Y, Li FF, Yang Z, Liu Y (2015). Cathepsin B deficiency attenuates cardiac remodeling in response to pressure overload via TNF-alpha/ASK1/JNK pathway. Am J Physiol Heart Circ Physiol.

[74] Tanaka T, Warner BM, Michael DG, Nakamura H, Odani T, Yin H (2022). LAMP3 inhibits autophagy and contributes to cell death by lysosomal membrane permeabilization. Autophagy.

[75] Edwan JH, Goldbach-Mansky R, Colbert RA (2015). Identification of interleukin-1beta-producing monocytes that are susceptible to pyronecrotic cell death in patients with neonatal-onset multisystem inflammatory disease. Arthritis Rheumatol.

[76] Saito M, Nishikomori R, Kambe N, Fujisawa A, Tanizaki H, Takeichi K (2008). Disease-associated CIAS1 mutations induce monocyte death, revealing low-level mosaicism in mutation-negative cryopyrin-associated periodic syndrome patients. Blood.

[77] Fujisawa A, Kambe N, Saito M, Nishikomori R, Tanizaki H, Kanazawa N (2007). Disease-associated mutations in CIAS1 induce cathepsin B-dependent rapid cell death of human THP-1 monocytic cells. Blood.

[78] Guicciardi ME, Deussing J, Miyoshi H, Bronk SF, Svingen PA, Peters C (2000). Cathepsin B contributes to TNF-alpha-mediated hepatocyte apoptosis by promoting mitochondrial release of cytochrome c. J Clin Invest.

[79] Li Z, Berk M, McIntyre TM, Gores GJ, Feldstein AE (2008). The lysosomal-mitochondrial axis in free fatty acid-induced hepatic lipotoxicity. Hepatology.

[80] Talukdar R, Sareen A, Zhu H, Yuan Z, Dixit A, Cheema H (2016). Release of cathepsin B in cytosol causes cell death in acute pancreatitis. Gastroenterology.

[81] Gong F, Peng X, Luo C, Shen G, Zhao C, Zou L (2013). Cathepsin B as a potential prognostic and therapeutic marker for human lung squamous cell carcinoma. Mol Cancer.

[82] Colin C, Voutsinos-Porche B, Nanni I, Fina F, Metellus P, Intagliata D (2009). High expression of cathepsin B and plasminogen activator inhibitor type-1 are strong predictors of survival in glioblastomas. Acta Neuropathol.

[83] Pandey G, Bakhshi S, Kumar M, Thakur B, Jain P, Kaur P (2019). Prognostic and therapeutic relevance of cathepsin B in pediatric acute myeloid leukemia. Am J Cancer Res.

[84] Wu JS, Li ZF, Wang HF, Yu XH, Pang X, Wu JB (2019). Cathepsin B defines leader cells during the collective invasion of salivary adenoid cystic carcinoma. Int J Oncol.

[85] Mijanovic O, Brankovic A, Panin AN, Savchuk S, Timashev P, Ulasov I (2019). Cathepsin B: a sellsword of cancer progression. Cancer Lett.

[86] Yanamandra N, Gumidyala KV, Waldron KG, Gujrati M, Olivero WC, Dinh DH (2004). Blockade of cathepsin B expression in human glioblastoma cells is associated with suppression of angiogenesis. Oncogene.

[87] Withana NP, Blum G, Sameni M, Slaney C, Anbalagan A, Olive MB (2012). Cathepsin B inhibition limits bone metastasis in breast cancer. Cancer Res.

[88] Victor BC, Anbalagan A, Mohamed MM, Sloane BF, Cavallo-Medved D (2011). Inhibition of cathepsin B activity attenuates extracellular matrix degradation and inflammatory breast cancer invasion. Breast Cancer Res.

[89] Wang K, Liu X, Liu Q, Ho IH, Wei X, Yin T (2020). Hederagenin potentiated cisplatin- and paclitaxel-mediated cytotoxicity by impairing autophagy in lung cancer cells. Cell Death Dis.

[90] Brun S, Bestion E, Raymond E, Bassissi F, Jilkova ZM, Mezouar S (2022). GNS561, a clinical-stage PPT1 inhibitor, is efficient against hepatocellular carcinoma via modulation of lysosomal functions. Autophagy.

[91] Meyer N, Henkel L, Linder B, Zielke S, Tascher G, Trautmann S (2021). Autophagy activation, lipotoxicity and lysosomal membrane permeabilization synergize to promote pimozide- and loperamide-induced glioma cell death. Autophagy.

[92] Watson CJ, Kreuzaler PA (2009). The role of cathepsins in involution and breast cancer. J Mammary Gland Biol Neoplasia.

[93] Kos J, Mitrovic A, Mirkovic B (2014). The current stage of cathepsin B inhibitors as potential anticancer agents. Future Med Chem.

[94] Aggarwal N, Sloane BF (2014). Cathepsin B: multiple roles in cancer. Proteom Clin Appl.

[95] Rudzinska-Radecka M, Frolova AS, Balakireva AV, Gorokhovets NV, Pokrovsky VS, Sokolova DV, et al. In silico, in vitro, and clinical investigations of cathepsin B and stefin A mRNA expression and a correlation analysis in kidney cancer. Cells. 2022;11. 10.3390/cells11091455. 10.3390/cells11091455 PMC9101197 35563761

[96] Simoes PSR, Zanelatto AO, Assis MC, Varella PPV, Yacubian EM, Carrete H (2019). Plasma kallikrein-kinin system contributes to peripheral inflammation in temporal lobe epilepsy. J Neurochem.

[97] Wadhawan M, Singh N, Rathaur S (2014). Inhibition of cathepsin B by E-64 induces oxidative stress and apoptosis in filarial parasite. PLoS ONE.

[98] Frlan R, Gobec S (2006). Inhibitors of cathepsin B. Curr Med Chem.

[99] Mazzotta C, Marden G, Farina A, Bujor A, Trojanowski MA, Trojanowska M (2021). FLI1 and ERG protein degradation is regulated via cathepsin B lysosomal pathway in human dermal microvascular endothelial cells. Microcirculation.

[100] Gan L, Ye S, Chu A, Anton K, Yi S, Vincent VA (2004). Identification of cathepsin B as a mediator of neuronal death induced by Abeta-activated microglial cells using a functional genomics approach. J Biol Chem.

[101] Wang J, Wang L, Zhang X, Xu Y, Chen L, Zhang W (2021). Cathepsin B aggravates acute pancreatitis by activating the NLRP3 inflammasome and promoting the caspase-1-induced pyroptosis. Int Immunopharmacol.

[102] Jia C, Zhang J, Chen H, Zhuge Y, Chen H, Qian F (2019). Endothelial cell pyroptosis plays an important role in Kawasaki disease via HMGB1/RAGE/cathespin B signaling pathway and NLRP3 inflammasome activation. Cell Death Dis.

[103] Schurigt U, Schad C, Glowa C, Baum U, Thomale K, Schnitzer JK (2010). Aziridine-2,3-dicarboxylate-based cysteine cathepsin inhibitors induce cell death in Leishmania major associated with accumulation of debris in autophagy-related lysosome-like vacuoles. Antimicrob Agents Chemother.

[104] Lyo V, Cattaruzza F, Kim TN, Walker AW, Paulick M, Cox D (2012). Active cathepsins B, L, and S in murine and human pancreatitis. Am J Physiol Gastrointest Liver Physiol.

[105] Mitrovic A, Mirkovic B, Sosic I, Gobec S, Kos J (2016). Inhibition of endopeptidase and exopeptidase activity of cathepsin B impairs extracellular matrix degradation and tumour invasion. Biol Chem.

[106] El-Fakharany EM, Abu-Serie MM, Habashy NH, Eltarahony M (2022). Augmenting apoptosis-mediated anticancer activity of lactoperoxidase and lactoferrin by nanocombination with copper and iron hybrid nanometals. Sci Rep.

[107] Dai Z, Cheng Q, Zhang Y (2020). Rational design of a humanized antibody inhibitor of cathepsin B. Biochemistry.

[108] Hook V, Yoon M, Mosier C, Ito G, Podvin S, Head BP (2020). Cathepsin B in neurodegeneration of Alzheimer’s disease, traumatic brain injury, and related brain disorders. Biochim Biophys Acta Proteins Proteom.

[109] Mitrovic A, Zavrsnik J, Mikhaylov G, Knez D, Pecar Fonovic U, Matjan Stefin P (2022). Evaluation of novel cathepsin-X inhibitors in vitro and in vivo and their ability to improve cathepsin-B-directed antitumor therapy. Cell Mol Life Sci.

[110] Unal-Cevik I, Kilinc M, Can A, Gursoy-Ozdemir Y, Dalkara T (2004). Apoptotic and necrotic death mechanisms are concomitantly activated in the same cell after cerebral ischemia. Stroke.

[111] Yuan D, Liu C, Wu J, Hu B (2018). Inactivation of NSF ATPase leads to cathepsin B release after transient cerebral ischemia. Transl Stroke Res.

[112] Gabande-Rodriguez E, Perez-Canamas A, Soto-Huelin B, Mitroi DN, Sanchez-Redondo S, Martinez-Saez E, et al. Lipid-induced lysosomal damage after demyelination corrupts microglia protective function in lysosomal storage disorders. EMBO J. 2019;38. 10.15252/embj.201899553. 10.15252/embj.201899553 PMC6331723 30530526

[113] Kingham PJ, Pocock JM (2001). Microglial secreted cathepsin B induces neuronal apoptosis. J Neurochem.

[114] Devika PT, Prince PS (2008). Preventive effect of (-)epigallocatechin-gallate (EGCG) on lysosomal enzymes in heart and subcellular fractions in isoproterenol-induced myocardial infarcted Wistar rats. Chem Biol Interact.

[115] Roy SJ, Stanely Mainzen Prince P (2012). Protective effects of sinapic acid on lysosomal dysfunction in isoproterenol induced myocardial infarcted rats. Food Chem Toxicol.

[116] Lambelet M, Terra LF, Fukaya M, Meyerovich K, Labriola L, Cardozo AK (2018). Dysfunctional autophagy following exposure to pro-inflammatory cytokines contributes to pancreatic beta-cell apoptosis. Cell Death Dis.

[117] Satoh T, Kambe N, Matsue H (2013). NLRP3 activation induces ASC-dependent programmed necrotic cell death, which leads to neutrophilic inflammation. Cell Death Dis.

[118] Ben-Ari Z, Mor E, Azarov D, Sulkes J, Tor R, Cheporko Y (2005). Cathepsin B inactivation attenuates the apoptotic injury induced by ischemia/reperfusion of mouse liver. Apoptosis.

[119] Maccioni L, Loriot A, Dewulf J, Bommer G, Horsmans Y, Lanthier N (2022). Duodenal CD8+ T resident memory cell apoptosis contributes to gut barrier dysfunction and microbial translocation in early alcohol-associated liver disease in humans. Aliment Pharm Ther.

[120] Kundu ST, Grzeskowiak CL, Fradette JJ, Gibson LA, Rodriguez LB, Creighton CJ (2018). TMEM106B drives lung cancer metastasis by inducing TFEB-dependent lysosome synthesis and secretion of cathepsins. Nat Commun.

[121] Feng L, Liang L, Zhang S, Yang J, Yue Y, Zhang X (2022). HMGB1 downregulation in retinal pigment epithelial cells protects against diabetic retinopathy through the autophagy-lysosome pathway. Autophagy.

[122] Jiang Y, Woosley AN, Sivalingam N, Natarajan S, Howe PH (2016). Cathepsin-B-mediated cleavage of Disabled-2 regulates TGF-beta-induced autophagy. Nat Cell Biol.

